# On Epidemic Cerebro-Spinal Meningitis, or Spotted Fever, with Cases

**Published:** 1865-02

**Authors:** John A. Liddell

**Affiliations:** Surgeon U.S. Vols.


### ON EPIDEMIC CEREBRO-SPINAL MENINGITIS, OR SPOTTED FEVER, WITH CASES.

BY JOHN A. LIDELL, M.D., Surgeon U.S. Vols.

A very formidable disease, designated by some as "*spotted fever*," and by others as "*epidemic cerebro-spinal meningitis*," has recently attracted a good deal of attention in professional circles. Of this, the pages of the leading medical journals of this country bear ample testimony. Besides, it has, within a few months past, been the theme of discussion at the meetings of some of the most influential and important of our medical societies; for example, the Philadelphia College of Physicians, and the New York Academy of Medicine. And, of a verity, no one can justly doubt that a disease so sudden in its attack, so rapid in its progress, so fatal in its results, and about which we really know so little, as the one under consideration, is worthy of all the attention which the best minds in the profession can bestow. It is also a subject which challenges the earnest attention of the humbler laborers in the cause of science and humanity; and, actuated by this belief, the writer desires to place the history of the following cases on record, as a contribution, although but a small one, more especially to our knowledge of the pathological anatomy of this obscure, erratic, and formidable disorder.

It should be premised, however, that these cases occurred recently among the inmates of Stanton General Hospital, Washington, D.C., that they occurred in portions of the hospital widely separated from each other, and that no relation by contact, whatever, can be traced between them. The first, in point of time, appeared in ward No. 8, July 28, the second in ward No. 3, July 31, and the third in the hospital guardhouse, August 14. For purposes of description, the second case in the order of time will be presented first, because its general features are more clearly defined than those pertaining to either of the others. It should be stated further, that these are the only cases of this disorder which have ever appeared at this hospital.

CASE I. *Cerebro-Spinal Meningitis.*—Private John Davis, Co. E, 8th Vermont Volunteers, 28 years old, and of a scrofulous diathesis, was admitted to Stanton General Hospital July 26, 1864, having an attack of intermittent fever, quotidian in type, and moderate in severity. He stated that, until recently, he had been serving in Gen. Banks' army, Department of the

Gulf. Quiniæ sulph. gr. v, every six hours, was prescribed for him, and a full diet was allowed. Under this treatment he improved rapidly, and his chills ceased after the first day. The quinia was continued, and he bade fair to return to duty speedily.

*July* 31, *morning.* He remained in bed, complaining of loss of appetite, and of feeling rather weak; his countenance expressed a good deal of anxiety, but there was no heat of skin, nor disturbance of the pulse, nor coating of the tongue. He said that, about midnight, he had been suddenly seized with great restlessness as he lay in bed. This lasted about twenty minutes, but he did not sleep till towards morning. He had no pain. The officer of the day being called, prescribed fluid extract of valerian for him in the night. At the morning visit he was also free from pain. The quinia was continued, and he was directed to keep his bed.

He got on well through the day until about 5½ o'clock P.M., when he was again attacked suddenly with great restlessness, and with irregular convulsive movements, closely resembling those presented by certain cases of hysteria; he became delirious, and uttered loud, shrill screams, at the same time tossing himself about very much, and throwing himself out of bed, unless restrained by the attendants; pupils dilated and symmetrical; no strabismus; pulse 100, and full, but there was no unnatural temperature of the skin; respirations about 14; no vomiting; could not be made to protrude his tongue, nor to swallow anything.

*Treatment.*—Ice was applied to the head and the spine, a sinapism was placed on the epigastrium, and a large turpentine enema was administered; the enema was repeated in an hour, but without effect in either case. No remedy was administered by the mouth, for the power of deglutition appeared to be lost.

*At* 7 *o'clock,* the pupils continued dilated; the respirations were 12; the skin was moist; the pulse 90; the restlessness diminished; but still he could not swallow.

*At* 9 *o'clock,* pupils dilated; respirations 12, but more labored; pulse 90, and weaker; less restlessness, and less screaming. After this, the screams were gradually replaced by low moans. He sank gradually into stupor, and died comatose at midnight, about 24 hours after the beginning of the attack, and about 6½ hours after its character was fully developed. Towards the last he was bathed in perspiration.

*Autopsy ten hours after death.*—Muscular system well developed; adipose tissue rather scanty; suggillations well marked in depending portions of cadaver; rigor mortis strong; scattered over the back of the neck and the dorsal aspect of the shoulders are 25 or 30 *dark purple spots*, circular in shape, somewhat raised above the surrounding skin, with a well-defined margin, and varying in size from a mustard-seed to a pea. On incising them, it is found that they present an infiltration of dark-colored blood in all the layers of the skin, and, to some extent, in the connective tissue beneath. The spots are not arranged in groups. On superficial view, they presented considerable resemblance to leech-bites.

*Cranium.*—While sawing off the calvarium, and, subsequently, while removing the brain, *a large quantity of serum* colored with blood flowed away. It amounted in all to at least *six ounces.* Glandulæ Pacchioni unusually numerous, large, and adherent, for a subject of only 28 years. A moderate quantity of limpid serum beneath the visceral arachnoid membrane, especially about the vertex. The arachnoid has lost its transparency and become more or less opaque (opacified), especially in the same locality. A moderate amount of limpid *serum* in the *ventricles.* The choroid plexus in the fourth ventricle is thickened, pale-red in color, and presents a striking resemblance to a lamina of pale, flabby granulations, but the choroid plexus in the other ventricles presents no abnormity. The cerebrum, cerebellum, pons Varolii, and medulla oblongata were carefully examined. They were moderately congested throughout, but presented no other abnormal appearance.

*Spinal canal.*—On sawing through the posterior vertebral arches (laminæ) and removing them so as to open the spinal canal almost its whole length, it was seen that the theca vertebralis was distended, or, at least, well filled with serum, notwithstanding the large amount of cerebro-spinal fluid which had escaped while examining the brain. On laying the theca open, it was found that the spinal arachnoid membrane had lost its smooth, shining appearance, and was opacified (pearl-colored), throughout its whole extent. The subarachnoidean bloodvessels were everywhere intensely congested (active hyperæmia). The cerebro-spinal fluid was noticed to contain some thin, white flocculi in the lumbar region; but otherwise it was limpid. The structure of the spinal cord did not present any abnormity.

*Thorax.*—No pleuritic adhesions; both lungs strongly congested (*passive hyperœmia*); an *apoplectic extravasation* of blood, as large as a walnut, was found in the middle lobe of the right lung, at the fissure between it and the superior lobe; there was a large cicatrix at apex of left lung, and, beneath it, a vomica inclosed by a thick membranous wall, and containing a quantity of whitish, cheese-like substance. The vomica was evidently undergoing contraction.* The heart exhibited rather more than the normal quantity of fat on its exterior, but in other respects was sound. The heart clots were small. The *blood* was much more *fluid* than natural, and a very large quantity of it flowed into the cavity of the chest on dividing the great vessels, while examining the thoracic organs. The abnormal fluidity of the blood constituted a marked feature of the case.

*Abdomen.*—The liver and intestines presented no abnormity. The kidneys were intensely congested, especially about the bases and cones of the pyramids, and presented a very dark-red, or almost brown color. The bladder was rather more than half full of amber-colored *urine*, which was found to be heavily loaded with *albumen*, on testing with heat and nitric acid.

*Remarks.*—The symptoms developed during life, and the pathological conditions revealed by the autopsy, show conclusively that the case belongs to the same category as those which have been described by numerous observers under the name of *cerebro-spinal meningitis.* On this point there is no room for a reasonable doubt. It is necessary, however, in taking a comprehensive view of this case, to observe that the pathological lesions were not confined to the organs located in the cranium and the spinal canal, but that the condition of the *blood* also was *markedly abnormal;* for it showed hardly any tendency to coagulate, remaining *preternaturally fluid* everywhere; while, at the same time, it manifested a very *strong tendency to extravasate*, which was denoted by the *pulmonary apoplexy* and by the *cutaneous purple spots.* It is, therefore, evident that the meningeal inflammatian did not constitute the whole of the case, by a great deal, and that the morbid process, or the disease itself, was not restricted to the cerebro-spinal axis and its meningeal investment. This view of the case is rendered still more probable by the fact that the kidneys were strongly congested, and that an abundant quantity of albumen was found in the *post mortem* urine.

As already stated, the patient died comatose. It might also be remarked, with perfect propriety, that he died asphyxiated, and in the following manner:—The respiration speedily sank to 12 per minute, while the pulse remained 90, and full. The heart continued to act energetically, and to force the blood with

* This was a tuberculous cavity in process of being cured, and was not only interesting *per se*, but also as occurring in a subject whose death was ultimately occasioned by another disorder.

abnormal rapidity into the lungs, while, at the same time, the respiratory movements were failing from increasing paralysis of the muscular apparatus by which these movements are accomplished. The blood, therefore, failing to be sufficiently decarbonized in the lungs, stagnated in those organs, and in this way was produced that passive hyperæmia, or pulmonary engorgement with venous blood, which was witnessed at the autopsy.

This motor paralysis of the muscles of respiration had a central origin, and was doubtless occasioned directly by mechanical compression of certain important nervous centres. The inflammatory process in the cerebro-spinal meninges was characterized by a very copious and rapid exudation of serum; so copious and rapid, indeed, that it might be styled an extravasation without any manifest impropriety. This serous fluid was, for the most part, poured out into the cavity of the arachnoid membrane. Some of it, indeed, was effused into the ventricles and into the sub-arachnoidean spaces, but the rest, and by far the largest part of it, was effused as already stated. This copious exudation of serum (about six ounces were found at the autopsy) in the cavity of the arachnoid membrane would tend to compress the brain and spinal cord alike. However this may be, it is certain that the organs located at the base of the brain and at the upper end of the spinal cord are very susceptible to pressure applied in this way. Thus it happened that the *functions of many nervous centres*, both sensitive and motor, were impaired, and finally *arrested*, by mechanical compression; and prominent among them was the *respiratory tract* of the medulla oblongata. Thus it happened that the patient lost the power to swallow from paralysis of the muscles of the pharynx, and the ability to breathe from paralysis of the muscles concerned in respiration; and he finally sank into stupor or coma, firstly, because the cerebral hemispheres were compressed, and, secondly, from the stupefying influence of the unaerated and highly carbonized blood which was circulating in the cerebral vessels.

The early death in this case, must, therefore, be attributed directly to the rapid exudation of a large quantity of serum into the cranio-vertebral cavity. Now, it is this wonderfully rapid and profuse outpouring of serum, which especially distinguishes the so-called epidemic cerebro-spinal meningitis from all other forms of inflammation of the membranes of the brain and spinal cord. For example, meningeal inflammation produced by violence exhibits an exudation of plastic lymph, or of pus, accompanied, for the most part, with but a small amount of serous effusion; and even in the cases of the so-called tubercular meningitis, the effusion of serum, although copious, always takes place much more slowly, and on that account life may sometimes be prolonged to an indefinite period, even when the amount of the effusion happens to be very great.

Very rapid and profuse effusion of serum is, therefore, a distinguishing feature of the kind of inflammation of the cerebro-spinal meninges now under consideration, and indicates that we must look beyond the membranes of the brain and spinal marrow, into the state of the system at large, if we expect to raise ourselves up to a comprehension of the subject. It stamps this form of meningitis as having a peculiar character, dependent, in all probability, upon a peculiar constitutional condition, which renders it as much a specific disease as erysipelas or rheumatism. The peculiarity of the erysipelatous dyscrasia is, that it predisposes to diffuse cellulo-cutaneous inflammation; of the rheumatic dyscrasia, that it induces fibro-articular inflammation; and the peculiarity of the unnamed dyscrasia which we are now considering is, that it is often associated with a meningeal inflammation, which is characterized by a sudden and copious effusion of serum.

CASE II. *Cerebral Meningitis with very Rapid Effusion.*—Private John Minsberger, Co. I, 90th Pennsylvania Volunteers, 40 years old, and sound in constitution, a patient at Stanton General Hospital, convalescent from resection of the left elbow-joint, had recovered so far that the wound of operation was healed, and he appeared to be in good health in every respect, having regained his flesh and strength.

On the morning of July 28th he arose, dressed himself, and went out to the pump for a drink of fresh water, as was his daily practice, and then returned to his ward, being, to all appearance, in usual health.

*About* 6 *o'clock* he was suddenly seized with a great pain in his back, between the shoulder-blades. He said it felt as if somebody was pressing a bar of hot iron into his backbone. A mustard plaster was applied, and he obtained speedy relief. He had no chill, and the attack appeared to begin without warning. After it was over, he got up and walked about the ward.

*About* 7 *o'clock* the paroxysm of burning pain returned; the sinapism was again applied, and he got relief in ten or fifteen minutes. He appeared to be sick at the stomach, and tried to vomit. His strength appeared to be good. He declined food, and had no thirst. This paroxysm was somewhat longer than the other. After it was over, he fell into a profuse perspiration, and slept an hour or two.

*About* 10 *o'clock* he was seized the third time with the same intense burning pain in the dorsal region, which he referred to the backbone. This paroxysm was much severer than the others, and he cried out for anguish. It also lasted much longer. There were no convulsive movements. The medical officer in charge of him, Acting Assistant-Surgeon W. B. Dick, U.S. Army, reported the case to me, stating that there did not seem to be much the matter with him during the intervals between the paroxysms, and I advised cupping of the affected part.

*At* 11 *o'clock*, on raising him up in bed for the cups to be applied, he was seized with the fourth paroxysm. He fell over on the shoulder of an attendant at once, exclaiming, "Oh, such pain!" and immediately became unconscious, as if in a syncope. At the same time his face became deathly pale, his eyes fixed, the muscular system relaxed, the radial pulse weak and fluttering, and the respiration was suspended, or, rather, he breathed at very long intervals only. I was hastily summoned, and in a few minutes saw him. The pallor of the face and lips had then given place to the livid hue of asphyxia; the eyes were open, fixed, and glassy, the right pupil was dilated, while the left was contracted; the muscular system was completely relaxed; no radial pulse was perceptible, and he took afterwards only three or four long, sighing inspirations, with long intervals between each of them, notwithstanding that ammonia was applied to the nostrils, and Marshall Hall's method of artificial respiration was faithfully tried. He breathed the last time about 11½ o'clock. His lips and face were then very livid. He had no thirst and no unnatural warmth of skin during any part of the five and a-half hours his sickness lasted. He did not exhibit petechiæ or any other kind of spots.

*Autopsy by Assist.-Surg. George A. Mursick, U.S. Vol., six hours after death.*—Body well developed; no emaciation; no rigor mortis. Upon removing the calvarium, about 4 oz. of blood and serum flowed out. The veins and sinus of the brain were congested with fluid blood. There was a moderate amount of subarachnoid effusion over the hemispheres, and the ventricles contained about an ounce of serum. The spinal canal was opened from the occiput to the sacrum. The spinal cord and its membranes appeared healthy, and there was no congestion of them.

*Thorax.*—The lungs were intensely congested with venous blood; otherwise healthy. There were about 8 oz. of serum contained in both pleural cavities. The pericardium contained about 2 oz. of fluid. The heart was slightly hypertrophied; the mitral valves were slightly thickened, the tricuspids were a good deal thickened by a new deposit between the folds of the endocardium. The aortic and pulmonary valves were normal. There was no insufficiency of any of the valves.

*Abdomen.*—All the abdominal viscera were healthy, except the kidneys; they were in a state of acute congestion, and of a bright maroon color. The urine contained albumen; it was obtained at the autopsy.

*Blood.*—There was no coagulation of the blood. It remained fluid everywhere, was dark in color, and flowed freely wherever an incision was made into the body.

*Remarks.*—While perusing the account of the *post mortem* examination of this case, it is probable that the reader has not failed to notice a strong similarity between the anatomico-pathological lesions presented by it and by the preceding case. In both of them a large amount of serous effusion was found in the cranio-spinal cavity, the lungs were engorged with venous blood, the kidneys were congested, the urine contained albumen, and the blood presented a remarkable fluidity in every part of the body. It will thus be perceived that the morbid process was essentially the same in both instances, and that, however much they may differ in other and minor particulars, it was the same disease which brought both of these men to an untimely death.

Among the points of dissimilarity we may notice, first, that in the case under consideration the effusion of serium upon the cerebro-spinal axis and into the ventricles of the brain was much more sudden and rapid than in the preceding case. Indeed, it was so sudden and rapid as to produce "shock." It caused him to fall over on the shoulder of an attendant, and immediately become unconscious, as if in a syncope. At the same time a deathly pallor overspread his countenance. Subsequently his face became livid, in consequence of pulmonary congestion, and in a short time he expired, the respiratory function having failed the first of all. Here is presented before us the whole of that group of fatal symptoms which Dr. Watson and others have described under the title of serous apoplexy. Now, the term serous apoplexy is, doubtless, well enough, so far as it goes. It expresses exactly the way in which death took place in this case, but it gives no idea whatever concerning the morbid process at large, which occasioned the abnormal fluidity of the blood, the albuminous urine, and the tendency to serous extravasations, which were developed in this patient. It signifies that the patient was stricken down, as by a blow, from sudden effusion of serum upon the great nervous centres, and thus denotes or represents a peculiarity of the particular instance of disease, rather than the disease itself, considered as an entity.

Another point of marked dissimilarity was exhibited in the clinical history. Minsberger complained, at short intervals, of suffering intense pain. He expressed himself as feeling, at times, as if a bar of hot iron was being pressed into his backbone, but had no convulsive movements. Davis, on the other hand, did not complain of pain, but had convulsive movements. But, as heretofore stated, the essential features of both cases present a great similarity to each other.

Case III. *Meningitis.*—Private Simeon Bond, 37th Co., 2d Battalion V.R.C., aged 25, and of feeble constitution, was admitted to Ward No. 7, Stanton General Hospital, Acting Assistant-Surgeon W. B. Dick, U.S.A., on Sunday evening, August 14, 1864, from the quarters of the hospital guard, to which he belonged. He had been unwell for several days, complaining of debility and loss of appetite, and the officer of the day had excused him from duty on that account. He had not been confined to bed. The orderly sergeant thought him to be slightly out of his head on the day before he entered the hospital (Saturday), and noticed that he ate nothing.

When admitted to the hospital, he was feverish, thirsty, and sick at the stomach, but did not vomit; said he had headache, and felt sick and weak; did not have chills, nor complain of any other pain; pulse about 90. Prescribed the neutral mixture and aconite. That night he was restless, and slept but little.

*Monday*, 15*th*. He was light-headed, and did not seem to realise where he was; he wanted to get up and steal away, but was quiet withal. His head was hot, his eyes somewhat injected, his tongue furred, and his pulse 95, and stronger. Ordered the ice-bag to the head, a brisk purge (pil. cathartic. comp. No. 3), and spiritus mindereri in combination with vin. antimonial. and nitric ether. A dose of morphia was administered in the evening, and he passed a better night. He had pain in the head, but none whatever in the spine or any other part of the body.

*Tuesday*, 16*th*. Patient lies in a stupor, from which, however, he can be roused without much difficulty; pupils somewhat dilated, and symmetrical; eyes more injected; pulse about 80, and full; respiration slower than natural, but deep and regular; has mild delirium, and complains of headache, but sinks back into the stupor again as soon as the disturbance ceases; has no spasms of any kind. Ordered the hair to be cropped short; emplast. cantharid., 4 by 6, to nape of neck; sinapisms to the epigastrium and to the inner side of his thighs; and quinia in full doses. His bowels had moved freely.

He did not improve; the stupor grew more profound, and he died comatose that night, about 11 o'clock, something more than 48 hours after he entered the hospital.

No autopsy, for the body was sent away next morning at daylight, for interment at a distance, before it could be conveniently examined. No spots on his body were noticed. The febrile movement was well marked. He did not have any convulsions. There was no symptons whatever indicative of a spinal lesion. The symptoms all pointed to a meningo-cerebral lesion.

*Pathology.*—On looking into the recent literature of the subject, the writer finds that the anatomical lesions revealed by *post mortem* examination of the cases which he has narrated bear a striking resemblance to, or, indeed, are almost identical with, those presented by Dr. Jewell's case of "*spotted fever*," which is reported in the Transactions of the Philadelphia College of Physicians (vide *American Journal of the American Sciences*, pp. 130, 131, July, 1864). This patient was a male, aged nine years, and died comatose after a sickness of but 48 hours' duration. He had vomiting, headache, dilated pupils, convulsive movements, and petechiæ during life. At the autopsy, made 24 hours after death, the vessels of the dura mater were found to be markedly congested with fluid blood; there was a copious effusion of serous fluid in the subarachnoid spaces and in the ventricles of the brain; there were about two ounces of serum in the pericardium; heart-clots small, very black, softish, and not unlike currant-jelly. The kidneys were strongly congested. The blood in the cardiac veins, in the mesenteric veins, and, indeed, in every part of the body examined, was abnormally fluid. There were some purpurous spots here and there upon the mesentery; and on incising one of the cutaneous petechiæ, it was found to consist of an ecchymosis in the substance of the true skin, and not of a mere effusion beneath the cuticle.

The *pathological lesion* most constantly presented by the disease under consideration appears to be that of the blood. It was exhibited in a marked degree by the several cases cited, and presented a singular uniformity in all of them. No matter whether the disease ran its course in five and one-half hours or two days, the blood retained a remarkable fluidity and a dark red color after death in both alike. No so, however, with the lesion of the cerebro-spinal meningis. In Case I. we found that the membranes of both the brain and the spinal chord were involved in the inflammatory process. In Case II. the *post mortem* traces of inflammatory action were confined to the brain alone. In Case III., also, the inflammatory lesion, as denoted by the clinical history, affected the brain and its membranes alone. In Dr. Jewell's case, just now referred to, both the brain and the spinal marrow were involved.

But this remarkable disorder may even march on to a fatal termination without the occurrence of inflammation (active hyperæmia) in any part of the cerebro-spinal meninges, as happened in Dr. Levick's case of "*spotted fever*," which is reported in the Transactions of the Philadelphia College of Physicians (vide *American Journal of the Medical Sciences*, p. 136, July, 1864). This patient was but 18 months old, and died after being sick but 14 hours. The case exhibited a very strong hemorrhagic tendency. The autopsy was made 25 hours after death, and showed that the surface of the body was everywhere mottled, that there were vibices on the knees, and petechiæ on the legs; that the vessels of the dura mater were filled with dark fluid blood; that the substance of the brain and medulla oblongata was natural in appearance and consistence; *that there was no effusion in the ventricles, and the most careful examination failed to detect the slightest evidence of inflammatory exudation.* As in the other cases to which reference has been made, the blood presented a remarkable fluidity in every part submitted to examination. The lungs contained a large quantity of fluid blood. The left ventricle of the heart contained two soft clots, each about the size of a pea. The right ventricle did not contain any clots, and the blood therein was thin and fluid, looking not unlike claret wine. Numerous extravasations of blood were also found in various parts of the body. A large ecchymosis was discovered beneath the pericranium, near the saggital suture. The intestines were everywhere dotted with minute extravasations of blood, both on their outer and inner surface. Similar ecchymoses were found on the bladder, in the kidneys, and on the diaphragm. In all the other cases we found an abnormal exudation of serum associated with inflammatory action in some part of the cerebro spinal meninges. In the case of this infant we did not find any cerebro-spinal inflammation whatever; but, in place of exudation of serum, there were numerous and remarkable extravasations of the blood itself, discovered in various parts of the body. It is not improbable that many cases of the so-called purpura hemorrhagica, especially those occurring during the infantile period of life, and being accompanied with febrile movement, belong in reality to the same category as the disease under consideration. Indeed, the writer himself is inclined to believe that he has seen some cases of this sort in time past.

Is the so-called "*spotted fever*" a form of *typhus petechialis?* Are they cognate diseases? It is true they bear considerable resemblance to each other in two important particulars, viz., both of them are blood diseases, and both of them are apt to be associated with cutaneous ecchymosis (petechiæ). But here the resemblance ends. If we examine their clinical history with a critical eye, we find that they differ from each other as widely as typhoid fever differs from measles. For example, spotted fever often runs its course in a few hours; typhus requires at least several days. Spotted fever is frequently attended with convulsive movements; typhus is never so accompanied. Spotted fever patients often die very suddenly and unexpectedly from coma and asphyxia, the *modus operandi* of which has already been explained; typhus patients do not die in this way. The eruption in spotted fever frequently appears on the first day, as it did in the case of Davis, also in the case of the young lad reported by Dr. Jewell, and in the case of the young child reported by Dr. Levick; while in typhus the eruption does not appear till the end of a week or more from the commencement of the prodromic symptoms. Thus clinical observation shows not only that these two diseases are not identical, but that they are in reality entirely distinct from each other.

What then is the true nature of the disorder under discussion? This question does not admit of a complete answer in the present state of our knowledge; but, at the same time, as has already been shown in this paper, there is good reason to believe that the so-called spotted fever, or epidemic cerebro-spinal meningitis, is a *specific disease* dependent upon a specific dyscrasia; that it is also a *blood-disease*, in which respect it is analagous to all other dyscrasic disorders; and that it is often accompanied with a sudden and copious exudation of serum into the cranio-vertebral cavity, or with extensive extravasations of the blood itself in various parts of the organism; *vide* the pulmonary apoplexy in the case of Davis, and the numerous and extensive ecchymoses in Dr. Levick's case.

In the course of the discussion on cerebro-spinal meningitis before the New York Academy of Medicine, April 20, 1864, Prof. Clark stated that he "was inclined to doubt whether the right name had been found for the disease; in some cases the brain and spinal cord were involved in the inflammation, and so far the term cerebro-spinal meningitis was correct enough; but in other cases the inflammation was limited to the brain; while in still other cases the brain and cord escaped altogether, and the inflammation spent its force upon the pericardium, pleura, and even upon the lungs. That being the case, the disease, in his opinion, was due to a condition of the system in which there was a tendency to inflammation, and the inflammation might show itself in one part of the body or another, dependent upon circumstances which we cannot at first appreciate." (Vide *American Medical Times*, May 14, 1864, p. 237.)

The results of our investigations strongly corroborate the views of Prof. Clark. For we found in Case I. that the disease manifested itself locally, by producing an inflammation of the arachnoid membrane of both the brain and spinal cord; in Case II. that the disease expended its force mainly on the cerebral arachnoid alone; in Case III. that the disease made its advent by impairing first the functions of the brain as the organ of the mind, subsequent to which fatal cerebral meningitis supervened, the spinal marrow being not involved in any way; and in Dr. Levick's case, that the disease expended its force in producing extravasations of blood beneath the pericranium, the diaphragmatic pleura, in the coats of the intestines, in the kidneys, and on the bladder, there being no inflammatory action whatever about the brain, spinal cord, or any other organ in the body. The conclusion is irresistible that the disease itself consists of a pathological condition of the system at large, whereof the various local lesions enumerated above are but the accidental manifestations. It has also been conclusively shown that this morbid condition of the system is accompanied by a strongly marked pathological condition of the blood, the most striking feature of which is, that its coagulability is almost entirely destroyed.

Is this disorder communicable from one person to another? On this point the evidence furnished by our observations is entirely negative in character. As already stated, each of these cases occurred in a distinct portion of the hospital, and no relation by contact can be traced between them. The disease did not spread to the other patients occupying the same ward, or the same quarters, in a single instance. But this fact does not by any means prove the disease to be uncommunicable, for it should be borne in mind that these hospital wards are as thoroughly ventilated, clean, and wholesome as our present knowledge of hygiene enables us to make them. It cannot be expected that diseases which are not strongly contagious will spread in such a place and under such circumstances. At the same time the writer does not doubt that, under circumstances favoring its development, the disease may radiate from the localities in which it first appears, and thus conform to one of the laws regulating the spread of infectious disorders in general; and he also does not doubt that, under favoring circumstances, it may be communicated from one person to another.

The cases which came under our observation were probably produced by some kind of an epidemic influence, which we are not able to appreciate with our present lack of knowledge concerning this disease, as well as the occult causation of epidemic diseases in general. The *influenza* did not prevail at the time; indeed, there was not a case of influenza then in the hospital. This fact is mentioned because some have been inclined to think that spotted fever is only and exalted form of influenza.

While speaking of the epidemic origin of the disease, as it appeared at Stanton Hospital, it is worthy of remark that our first case, in point of time, occurred July 28th, and terminated fatally in five and one-half hours; that our second case occurred three days afterwards, July 31st, and terminated fatally in twenty-four hours; and that our third case died August 16th, after three days' sickness. This progressive diminution in the severity of type of the disorder from the first to the last case is either a remarkable coincidence, or (what is more probable) it shows that the special zymosis which produced the disease in question was also progressively diminishing in point of intensity.

*Treatment.*—Candor compels us to state that the remedial measures employed in our cases were not productive of much benefit. Davis lost the power of deglutition so early that the range of medication in his case was very much restricted. Even the stimulating enema failed to operate, apparently from paralysis of the muscular coat of the large intestine; and when it was repeated a short time afterwards there was still no effect, in all probability for the same reason. There is no reason to believe that the ice-bags applied to his head and spine did any good. In the case of Minsberger, the counter-irritation (sinapisms) seemed to afford some relief from the excruciating pain between his shoulder-blades; but, at the same time, it is not by any means certain that his paroxysms of pain were either shortened in duration or moderated in severity by these applications. In the case of Bond, neither the brisk purge, nor the aconite, nor the antimony, nor the blister on the neck appeared to have any effect in retarding the onward progress of the disorder.

The only remedy which seemed to benefit him was morphia, which, administered in the evening, enabled him to pass a better night. But notwithstanding this, he also, like the others, finally sank into stupor and died comatose. This analysis of the treatment employed in these cases has been made for the purpose of illustrating an instructive part of their clinical history.

All of these patients died from effusion of serum upon some part of the cerebro-spinal axis. Now, any plan of treatment, to have been successful, must either have prevented the effusion of the serum, or caused its absorption prior to the occurrence of fatal compression of the nervous centres. The remedies which we employed did not accomplish either of these desirable results. The question next arises whether there is any therapeutic agent at our disposal suitable for such a purpose. In the opinion of the writer we have such a remedy, and it is opium. It has for a considerable time been well known to many persons that opium, especially when administered in large doses, possessed a marvellous power in the way of arresting exudation from serous membranes. This property of opium has probably been most strikingly exhibited in the treatment of peritonitis with a suppurative tendency; for example, puerperal peritonitis; but its efficacy has also been fully proved in the management of inflammations of the thoraric serous membranes. But at a time like the present, when opium or its chief alkaloid is so extensively and successfully employed in surgical practice for the cure of traumatic peritonitis, it is not necessary to say any more on this point. And, entertaining these views, the writer is fully determined to give opium a fair trial in the treatment of spotted fever, if it should ever be his misfortune to again encounter that formidable disorder.

But the employment of opium in the treatment of epidemic cerebro-spinal meningitis is no new thing. Dr. Miner long ago strongly recommended its use in large doses. He said: "A few cases imperiously required half an ounce of the tincture of opium in an hour, or half a drachm in substance in the course of twelve hours, before urgent symptoms could be controlled; and even some cases required a drachm in the same time. All those patients whose symptoms were promptly met with opium invariably recovered." (Vide *Miner on Typhus Syncopalis.*)

According to Dr. Hartshorne: "The only treatment in which, after considerable experience, Boudin placed any confidence was that in which opium was used remedially. An *opiate sleep* was often followed by convalescence." (Vide *Am. Jour. of Med. Sciences*, p. 94, July, 1864.)

Not long since Dr. Walter F. Atlee treated ten cases of epidemic cerebro-spinal meningitis at St. Vincent's Home, in Philadelphia, with gratifying success, although nine of them were young children from two to four years of age. The treatment consisted in the application of irritants to the back, and in the administration of opium, the patients' strength being supported at the same time with brandy, wine-whey, and essence of beef. (*Op. cit.*, p. 95, July, 1864.)

Some have expressed a great deal of confidence in the efficacy of sulphate of quinia administered in large doses. But according to our experience, however useful this remedy may be for the removal of a malarial complication, it exerts but little if any influence upon the disease itself. For in the case of Davis the disease appeared in a very severe form while he was taking five grains of quinia three times a-day, and in the case of Bond large doses of that remedy did not produce any perceptible effect whatever.—*Am. Jour. Med. Sciences.*

